# Heterocycles 48. Synthesis, Characterization and Biological Evaluation of Imidazo[2,1-*b*][1,3,4]Thiadiazole Derivatives as Anti-Inflammatory Agents

**DOI:** 10.3390/molecules23102425

**Published:** 2018-09-21

**Authors:** Anamaria Cristina, Denisa Leonte, Laurian Vlase, László Csaba Bencze, Silvia Imre, Gabriel Marc, Bogdan Apan, Cristina Mogoșan, Valentin Zaharia

**Affiliations:** 1 Department of Organic Chemistry, Iuliu Hațieganu University of Medicine and Pharmacy, Cluj-Napoca 400012, Romania; anamaria.cristina@umfcluj.ro (A.C.); hapau.denisa@umfcluj.ro (D.L.); 2 Department of Pharmacology, Physiology and Pathophysiology, Iuliu Hațieganu University of Medicine and Pharmacy, Cluj-Napoca 400349, Romania; 3 Department of Pharmaceutical Technology and Biopharmaceutics, Iuliu Hațieganu University of Medicine and Pharmacy, Cluj-Napoca 400012, Romania; laurian.vlase@umfcluj.ro; 4 Biocatalysis and Biotransformation Research Group, Babeș-Bolyai University, Cluj-Napoca 400028, Romania; cslbencze@yahoo.com; 5 Department of Analytical Chemistry and Drug Analysis, Tîrgu Mureș University of Medicine and Pharmacy, Târgu Mureș 540139, Romania; silsta@yahoo.com; 6 Department of Pharmaceutical Chemistry, Iuliu Hațieganu University of Medicine and Pharmacy, Cluj-Napoca 400012, Romania; marc.gabriel@umfcluj.ro; 7 Department of Pharmacology, Toxicology and Clinical Pharmacology, Iuliu Hațieganu University of Medicine and Pharmacy, Cluj-Napoca 400349, Romania; apan_bogdan@yahoo.com

**Keywords:** imidazo[2,1-*b*][1,3,4]thiadiazole derivatives, anti-inflammatory activity, molecular docking, antinociceptive activity

## Abstract

Non-steroidal anti-inflammatory drugs (NSAIDs) are an important pharmacological class of drugs used for the treatment of inflammatory diseases. They are also characterized by severe side effects, such as gastrointestinal damage, increased cardiovascular risk and renal function abnormalities. In order to synthesize new anti-inflammatory and analgesic compounds with a safer profile of side effects, a series of 2,6-diaryl-imidazo[2,1-*b*][1,3,4]thiadiazole derivatives **5a**–**l** were synthesized and evaluated in vivo for their anti-inflammatory and analgesic activities in carrageenan-induced rat paw edema. Among all compounds, **5c** showed better anti-inflammatory activity compared to diclofenac, the standard drug, and compounds **5g**, **5i**, **5j** presented a comparable antinociceptive activity to diclofenac. None of the compounds showed ulcerogenic activity. Molecular docking studies were carried out to investigate the theoretical bond interactions between the compounds and target, the cyclooxygenases (COX-1/COX-2). The compound **5c** exhibited a higher inhibition of COX-2 compared to diclofenac.

## 1. Introduction

Inflammation is a complex defensive process of the body which reflects the response of the organism to various noxious stimuli. In the inflammatory process, the body tissues are affected by heat, redness, swelling and pain [1]. Non-steroidal anti-inflammatory drugs (NSAIDs) are the most widely used therapeutics for different diseases characterized by fever, pain and inflammation. It is well known that NSAIDs inhibit prostaglandin-endoperoxide H synthase (PGHS) or cyclooxygenase (COX), in a non-selective or selective manner. There are three isoforms of COX, the most studied being COX-1 and COX-2. COX-1 is the constitutive enzyme and is expressed in most tissues, being responsible for the physiological production of prostaglandins, the protection of gastric mucosa, kidney function and platelet aggregation. COX-2 is the inducible isoform and is the product of an immediate early response gene in inflammatory cells [2,3,4]. Nevertheless, the long-term use of NSAIDs treatment is often limited by gastrointestinal complications, such as ulcerations and hemorrhages that may result from the suppression of physiological prostaglandins production in these tissues [2,5].

The ability of a selective COX-2 inhibitor to decrease prostaglandin production and acute tissue inflammation at dosages that do not affect the stomach prostaglandin production has been demonstrated. This fact suggests that COX-2 selective inhibitors may provide a safer therapeutic alternative to non-selective NSAIDs [2,6].

Considering this, we proposed the synthesis of new derivatives that inhibit COX-2 in a higher percentage than COX-1. To obtain this effect, we have chosen as reference molecule celecoxib, a COX-2 specific inhibitor. However, we have proposed the synthesis of compounds with COX-2 selective inhibition, but not COX-2 specific inhibition because of the high cardiovascular risk. Thus, for the comparison of the anti-inflammatory, analgesic and especially the ulcerogenic potential, we have selected diclofenac as drug reference, which is a non-selective NSAID, but also the most active against COX-2 compared to other NSAIDs carboxylic acids derivatives [6,7].

In recent years, imidazo[2,1-*b*][1,3,4]thiadiazole derivatives have been intensively studied because of their wide range of biological activities. The imidazo[2,1-*b*][1,3,4]thiadiazole scaffold makes up the core structure of pharmacological active molecules possessing antimicrobial [8,9], anticonvulsant [10,11], diuretic [12], anticancer [13,14,15], antitubercular [16], analgesic [17] and anti-inflammatory [18,19] properties.

**Figure 1:**
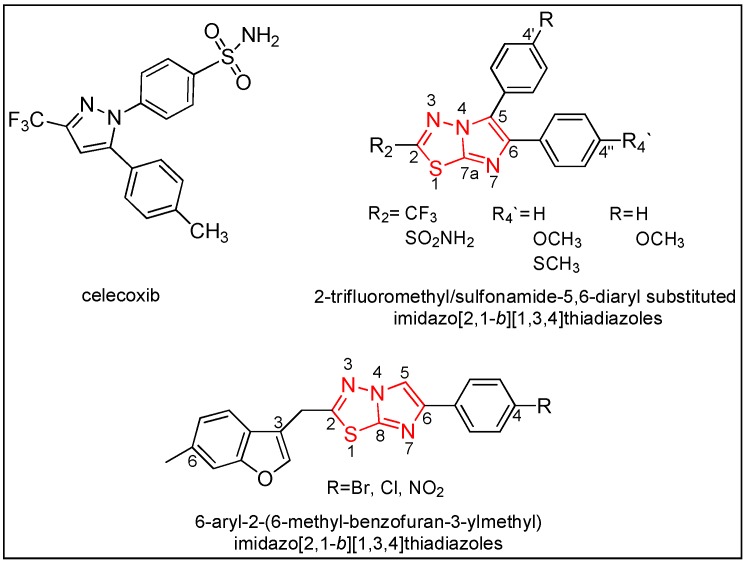
Structures of the literature lead compounds (Reproduced with permission from [Journal of Saudi Chemical Society]; published by [Elsevier]; No. 4425891193269).

Encouraged by the above literature and in continuation of our research work concerning the synthesis of diaryl-heterocyclic structures with anti-inflammatory and analgesic activities [20,21,22], we proposed the synthesis of 2,6-diaryl-imidazo[2,1-*b*][1,3,4]thiadiazole derivatives. We used as templates for our structures celecoxib and various imidazo[2,1-*b*][1,3,4]thiadiazoles (Figure 1) with anti-inflammatory potential: 6-carbamic acid-2,6-dimethylimidazo[2,1-*b*][1,3,4]thiadiazoles [17], 6-(hetero)arylimidazo[2,1-*b*][1,3,4]thiadiazole-2-sulfonamides [23], 2-trifluoromethyl/sulfonamide-5,6-diaryl substituted imidazo[2,1-*b*][1,3,4]thiadiazoles [19], 6-aryl-2-(6-methyl-benzofuran-3-ylmethyl)imidazo[2,1-*b*][1,3,4]thiadiazoles [18], 2,6-diaryl-imidazo[2,1-*b*][1,3,4] thiadiazoles [24].

The validity of the synthesized molecules was assessed through preliminary in vivo anti-inflammatory/analgesic screening and docking simulation, which highlighted the anti-inflammatory potential of the compounds.

## 2. Results

### 2.1. Chemistry

**Scheme 1:**
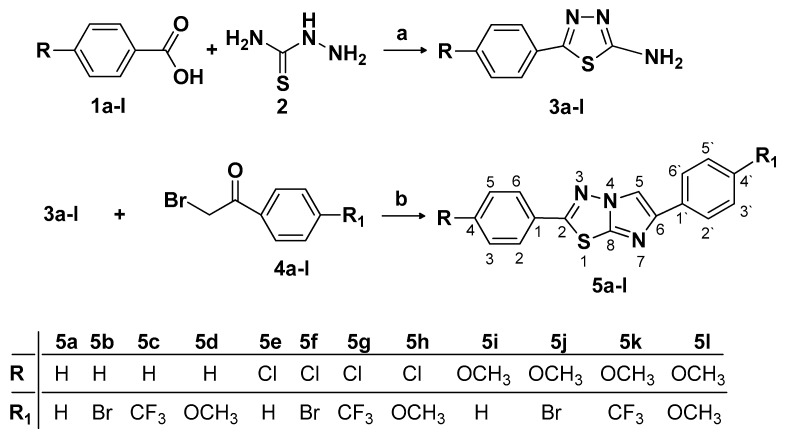
The synthesis of 2,6-substituted imidazo[2,1-*b*][1,3,4]thiadiazoles **5a**–**l**. (**a**) POCl_3_, reflux 2 h; H_2_O, reflux 4 h; (**b)** EtOH, reflux 18 h.

The synthesis route of 2,6-substituted imidazo[2,1-*b*][1,3,4]thiadiazoles **5a**–**l**, is illustrated in Scheme 1. The 6-(4-substituted phenyl)-2-(4-substituted phenyl)imidazo[2,1-*b*][1,3,4]thiadiazole **5a**–**l** was achieved in a one-pot reaction by the condensation of 5-(4-substituted phenyl)-1,3,4-thiadiazole-2-amine **3a**–**l** with substituted phenacyl bromide **4a**–**l**, under reflux in dry ethanol [19,25,26]. The 5-(4-substituted phenyl)-1,3,4-thiadiazole-2-amine **3a**–**l** was previously obtained by the cyclization of aromatic carboxylic acids **1a**–**l**, treated with thiosemicarbazide **2** in the presence of phosphorus oxytrichloride [9,20].

The synthesized compounds were purified and physico-chemically characterized by melting points (m.p.), infrared spectra (IR), nuclear magnetic resonance (^1^H-NMR, ^13^C-NMR and ^19^F-NMR) and mass spectrometry (MS). The details of the synthetic procedures, physico-chemical analysis and spectral data of the synthesized compounds are presented in the Materials and Methods section. Three of the compounds, **5i**, **5j**, **5l** were reported in literature as anticancer agents [26]. In addition, the compounds **5b**, **5f** and **5h** were reported in the literature possessing antibacterial (**5f**, **5h**) [8] and antifungal (**5b**, **5f**, **5h**) [8,27] properties.

The compounds **5b** [27,28], **5d** [16,28], **5h** [8,16,27], **5i** [26], **5j** [26], **5l** [26] were physico-chemical and spectral (IR, MS, ^1^H-NMR, ^13^C-NMR) characterized in the literature, while the compounds **5a [28]** and **5f [27,28]** were incomplete characterized.

The IR spectra of the synthesized compounds **5a**–**l** showed the characteristic =CH- stretching bands at 3125–2922 cm^−1^.

The ^1^H-NMR spectra of compounds **5a**–**l** displayed the characteristic singlet for C-5 proton of imidazo[2,1-*b*][1,3,4]thiadiazole at δ 8.93–7.89 ppm. The signals appearing around δ 4.06–3.19 ppm confirm the presence of OCH_3_ group from **5d**, **5h**, **5i**, **5j**, **5k**, **5l** compounds.

The ^13^C-NMR spectra of the compounds **5a**–**l** revealed the presence of the carbon atom from the 5th position of imidazo[2,1-*b*][1,3,4]thiadiazole at δ 116.28–100.25 ppm. The aliphatic signals of carbon atoms from OCH_3_ group (**5d**, **5h**, **5i**, **5j**, **5k**, **5l)** appeared at δ 56.82–55.33 ppm and those from CF_3_ group (**5c**, **5g**, **5k**) were observed at δ 127.09–126.9 ppm. In the ^19^F-NMR spectra of the compounds **5c**, **5g**, **5k**, the signal of the -CF_3_ group appeared at δ −64.05–64.03 ppm.

The MS spectra of the compounds **5b**, **5f** and **5j** with bromine atoms in their structure and of the compounds **5e**, **5f**, **5g**, **5h** with chlorine atoms, presented characteristic peaks corresponding to the two isotopes.

### 2.2. Anti-Inflammatory Activity

The 12 synthesized compounds **5a**–**l** and diclofenac, as reference drug, were tested for their in vivo anti-inflammatory activity by modified carrageenan-induced rat paw edema model, firstly described by Winter et al. [29]. The results of anti-inflammatory potential are shown in Table 1.

When compared to control negative group, all the synthesized compounds (**5a**–**l**) administered in doses of 50 mg/kg bw, by gavage, showed a reduction in edema volume. The group treated with diclofenac (20mg/kg, bw), the non-selective NSAID, presented a significant reduction of edema volume, especially at 2, 3 and 4 h after the λ-carrageenan administration. One hour after the inflammation induction, compounds **5b** (20.87%), **5c** (27.50%), **5e** (37.88%), **5h** (33.78%), **5i** (26.90%), **5j** (31.10%), **5l** (20.63%) showed better anti-inflammatory activity than diclofenac group, but the results were not statistically significant. At 2 and 3 h after the inflammation occurred, the compounds **5c** (31.63%, 24.95%) and **5b** (19.92%) significantly decreased paw edema. Compounds **5a** (24.40%), **5b** (25.60%), **5c** (27.53%), **5h** (28.05%), **5i** (26.59%) and **5j** (27.53%) showed a significant edema inhibition at 4 h after inflammation occurred and the compounds **5c**, **5h**, **5j** had an anti-inflammatory activity better than diclofenac (26.96%).

### 2.3. Antinociceptive Activity

The antinociceptive activity was evaluated on a model of inflammatory pain induced with λ-carrageenan in rats, by a modified method of Randall and Selitto [30,31]. The oral administration of the synthesized compounds **5a**–**l** in doses of 50 mg/kg bw, produced a good increase of nociceptive threshold when compared to negative control group. The results are presented in Table 2.

The induction of paw inflammation with λ-carrageenan produced a decrease in the nociceptive threshold as edema volume increased from 1 to 4 h. Compounds **5g**, **5i**, **5j** showed a significant increase in the nociceptive threshold when compared to control negative group. At 4 h after the intraplantar injection of λ-carrageenan, all the synthesized compounds, except **5a** and **5d**, presented a significant increase in nociception threshold and the compound **5g** (80.00 ± 5.16 g) induced a better antinociceptive action than diclofenac (78.33 ± 4.77 g).

### 2.4. Ulcerogenic Activity

All the compounds were evaluated for their ulcerogenic side effect after a single intragastric administration of 50 mg/kg bw in rats. After we isolated the stomach, we assessed the ulceration score by examining the gastric mucosa for irritation, ulcerations and microhemorrhages by a magnifying lens. The results are presented in Table 3.

All synthesized compounds have shown a reduced ulcerogenic activity, with the stomach ulceration score between 0.00 ± 0.00 and 0.42 ± 0.20, when compared to diclofenac. The standard drug showed a high score of 2.58 ± 0.27, inducing a significant ulceration of the stomach mucosa when compared to negative control group. The compound **5k** has shown a good safety profile, and none of the synthesized compounds induced significant ulceration.

### 2.5. Molecular Docking

The comparative molecular docking study of the compounds **5a**–**l** to the cyclooxygenases, COX-1 and COX-2, was carried out in order to clarify the binding mode of the compounds and to evaluate the affinity of the tested compounds to both isoforms. The results of the molecular docking study are presented in Table 4.

Overall, our series of compounds have a higher affinity to COX-2, than COX-1. Some compounds presented a higher affinity to COX-2, referred to diclofenac (**5a**, **5b**, **5c**, **5e**, **5h**, **5i** and **5j**). Among them, compounds **5c**, **5e**, **5h** and **5j**, seemed to have a higher selectivity for COX-2, expressed as a *K*i_COX2_/*K*i_COX1_ low ratio.

The clustering analysis is reported in the literature as a method of removal of the false positive results for the molecular docking study [32,33]. A compound with a high homogeneity in binding to the protein can be considered as a true binder. Thus, a compound with a high homogeneity has a high number of conformations in the cluster of the best binding pose and a low number of other wandering conformations in other clusters, including few other conformations. Approaching the idea in the opposite direction, a compound with many clusters resulted after the molecular docking and with few poses in the same cluster can be considered as a false binder, having a high dispersion between conformations given by the different docking runs with random beginning positions [32,33]. The clustering analysis of the resulted bounds characteristic for the compounds **5a**–**l** are presented in Table 5.

All tested compounds **5a**–**l** are predicted to bind to COX-1 in a homogenous manner. They have at least half of the predicted conformations in the same two. A root mean square deviation (RMSD) cluster with the conformation predicted to have the highest affinity. The same homogenous pattern in binding is found in the side of COX-2 too, with some exceptions (**5d** and **5e**), which both have a more chaotic binding to the enzyme. Overall, compounds in our series have a maximum of three binding clusters, suggesting homogenous binding to COX-1 and COX-2.

**Figure 2:**
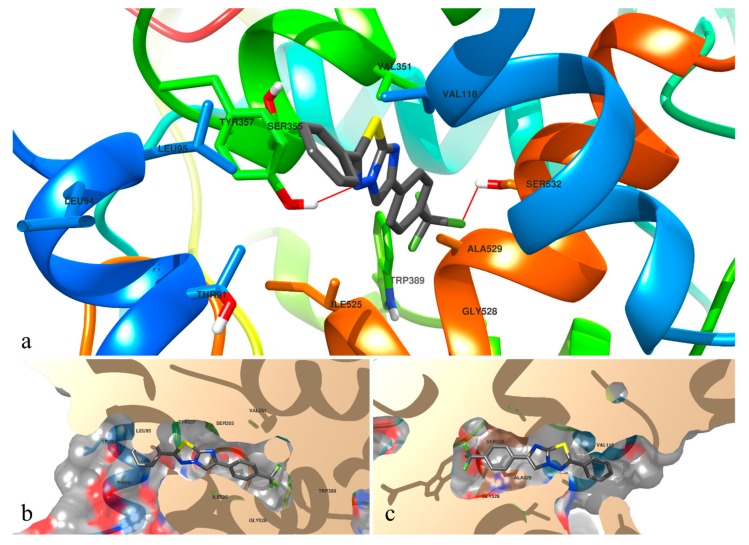
The top binding pose of compound **5c** docked to the catalytic site of COX-1. In section (**a**) of the figure, compound **5c** is depicted by segments, while the protein components are depicted in their tertiary structure, colored distinctly. In the next sections (**b**,**c**), the top binding pose of **5c** is depicted as in section (**a**), visualized from both sides, with the NSAID binding pocket of the COX-1 illustrated more realistically, using surfaces. Hydrogen bonds are depicted in red lines.

**Figure 3:**
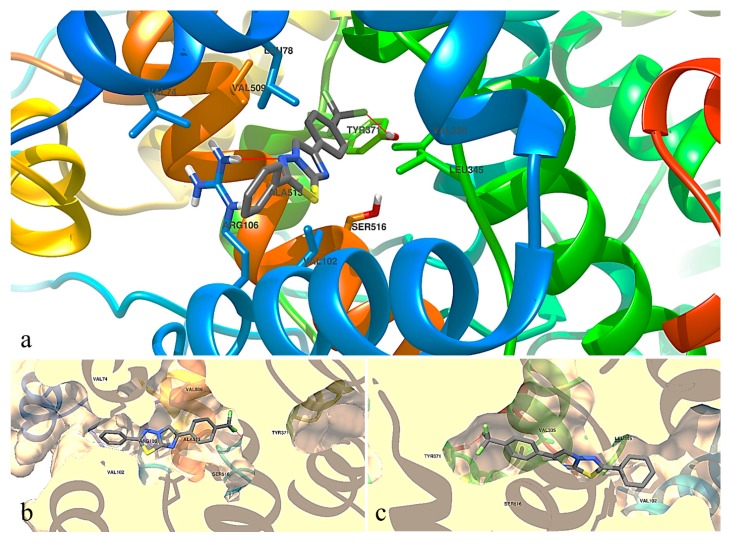
The top binding pose of compound **5c** docked to the catalytic site of COX-2. In section (**a**) of the figure, compound **5c** is depicted by segments, while the protein components are depicted in their tertiary structure, colored distinctly. In the next sections (**b**,**c**), the top binding pose of **5c** is depicted as in section (**a**), visualized from both sides, with the NSAID binding pocket of the COX-2 illustrated more realistically, using surfaces. Hydrogen bonds are depicted in red lines.

The analysis of the binding pattern showed that some amino acid residues are important in the formation of the hydrogen bond with the synthetic molecules. Based on the results we observed that Tyr357 and Ser523 are the most important polar amino acids from COX-1 and Ser516, Arg106 and Tyr371 from COX-2. The hydrogen bonds and the interaction diagrams for the synthetized compounds are shown in Table 6, and respectively in Figure 2 (interaction of **5c** with COX-1) and Figure 3 (interaction of **5c** with COX-2).

The substituents from the *para* position of the both aromatic rings are defining for the interaction between synthesized compounds in our series. These substituents influence the binding of the ligands to the protein, not especially because of the bonds between them and the protein, but by influencing the position of the ligand inside the pocket on the long axis of the ellipsoid which characterizes the NSAID binding pocket. The substituent from the *para* position, especially if it has a large volume (such as bromine **5b**, **5f**, **5j**), will tend to be oriented to the distal end of the NSAID binding pocket. This will influence the positioning of the heterocycle imidazo[2,1-*b*][1,3,4]-thiadiazole relative to Tyr387 in COX-1. The tyrosine residue is considered important in the literature reports, because it has a pivotal role in the hydrophobic environment [34]. Depending on the size of the substituents on the aromatic rings, it will lead to formation, or not, of a hydrogen bond with the nitrogen atoms from the imidazo[2,1-*b*][1,3,4]-thiadiazole fragment. An interesting aspect observed in our series is the presence of the CF_3_ substituent, which can form hydrogen bonds with Ser532 from COX-1 and Tyr371 from COX-2 and could influence the orientation of the ligand in the binding pocket.

**Figure 4:**
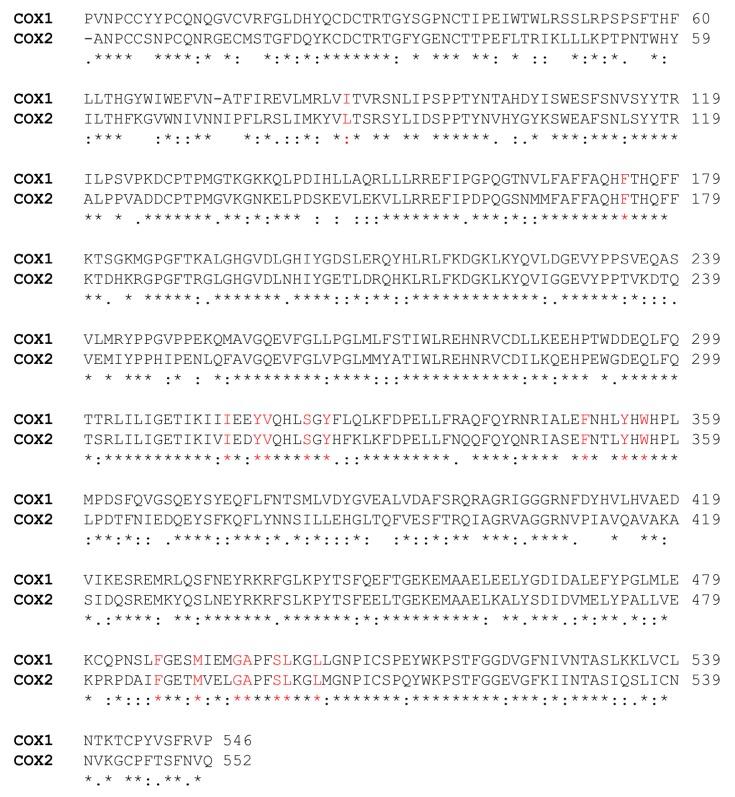
Pairwise sequence alignment between COX-1 and COX-2. The amino acids present in the catalytic site are depicted in red. Between the two proteins, large groups of identical amino acids were found, and the specified pairing is marked with an asterisk (*). An intermediate pairing, such as conservative substitutions between the two COX isozymes are depicted using a colon (:) or dot (.), while the lack of similarity was depicted as white space. The 17 catalytic amino acids provided by the blast analysis are depicted in red. The single difference in terms of amino acid substitution in the catalytic site between the two enzymes is the presence of Ile86 in COX-1, while in COX-2, at the same position, Leu86 can be found.

Paired sequence alignment between COX-1 and COX-2 brought an obvious similarity between these two isozymes, with a low evolutionary distance. The evaluation of the similarity of the amino acids after pairing was depicted underneath their alignment using graphical signs, depending on the degree of similarity between the two amino acids (Figure 4).

## 3. Discussion

Based on the molecules design reported in literature which is known to possess anti-inflammatory activity by blocking the COX-1/COX-2 [13,25], we synthesized and evaluated the anti-inflammatory and analgesic activity of 2,6-diaryl-imidazo[2,1-*b*][1,3,4]thiadiazole substituted with various pharmacophores. The synthesis of **5a**–**l** compounds was achieved by previously reported methods [19,25,26]. The structures of the synthesized compounds were assessed based on their spectral analysis: IR, ^1^H-NMR, ^13^C-NMR, ^19^F-NMR and MS. In the IR spectrum, the establishment of synthesized structures **5a**–**l** was carried out by the absence of NH_2_ bands around 3200 ppm. In addition, in ^1^H-NMR spectra of **5a**–**l**, we identified the presence of the characteristic signal of imidazole proton which confirms the formation of desired compounds. In the ^13^C-NMR spectra, all signals of the carbon atoms of the aromatic rings are present in the aromatic region and the aliphatic signals corresponding to the carbon atoms from the CF_3_ and OCH_3_ groups are also present.

The MS spectra confirmed the structures of the synthesized compounds **5a**–**l** by the presence of characteristic molecular peak.

The physico-chemical and spectral results obtained for the compounds **5a**, **5b**, **5d**, **5f**, **5h**, **5i**, **5j**, **5l** are in accordance with those reported previously in the literature [8,16,26,27,28].

All synthesized compounds were evaluated in vivo for their anti-inflammatory, analgesic and ulcerogenic potential and in silico (molecular modeling) for their interaction with cyclooxygenases.

The administration of carrageenan into the hind paw of rats, produces the cardinal signs of inflammation: edema, erythema and hyperalgesia [35]. It is known that the peripheral inflammatory response to carrageenan has a biphasic mechanism and if the edema inhibition is more evident in the initial edematous phase, the compounds mechanism of action is due to the inhibition of histamine and 5-hydroxytriptamine, followed by the inhibition of bradykinin release [1,35,36]. The second phase is attributed to an increase of prostaglandins in the damaged tissue [37,38,39]. The inflammatory effect of λ-carrageenan is explained by its interaction with Toll-like receptor 4 which induces the activation of Nuclear Factor κB (NFκB) and interleukin 8 (IL-8) through a pathway of innate immunity mediated by B-cell CLL/lymphoma 10 [40]. NF-κB is a major transcription factor for regulating the expressions of proinflammatory enzymes and cytokines (iNOS, COX-2 and TNF-α) [41]. COX-2 is an inducible enzyme and is responsible for the elevated production of prostaglandins (E2 and I2) during inflammation [4]. The NSAIDs mechanism of action is correlated with the non-selective inhibition of COX-1 and COX-2 isozymes [6]. In the first phase of edema development, at 1 h after the inflammation occurs, compounds **5b**, **5c**, **5d**, **5e**, **5f**, **5h**, **5i**, **5j** and **5l** presented an increased edema inhibition compared to negative control group and better than diclofenac. Then, at 4 h after the inflammation occurs, compounds **5a**, **5b**, **5c**, **5h**, **5i** and **5j**, have shown a significant inflammation decrease when compared to negative control group. In these compounds, we can observe that the significant reduction of edema volume was exceeded by those containing bromo (**5b**, **5j**), chloro (**5h**), trifluoromethyl (**5c**) and methoxy (**5h**, **5i**, **5j**) substituents. The compound **5c** seems to have the most significant edema inhibition, at 2, 3 and 4 h after inflammation induction, when compared to control negative group. The structure-activity relationship studies suggest that the presence of unsubstituted phenyl inhibits both COX-1 and COX-2 and the presence of methoxy and halogen groups at the 4th position of the 6-phenyl ring contributes for selective COX-2 inhibitory activity [19].

The molecular docking study was performed in order to compare the binding selectivity of the tested compounds to the catalytic sites of COX-1 and COX-2 and to observe if there is a correlation between in vivo and in silico studies of anti-inflammatory potential. Because of the closeness of these protein structures, development of novel agents with selective inhibition against COX-2 is challenging. Diclofenac binds to COX-1 and COX-2 through its carboxylate group hydrogen-bonded to Tyr387 and Ser532, respectively Tyr371 and Ser516, amino acids residues which are important to its anti-inflammatory activity [7].

The compound **5c** had the smallest *K*i_COX-2_/*K*i_COX-1_ in our series, which can be interpreted as having a higher selectivity to COX-2 instead of COX-1, compared to the other compounds. More than that, compound **5c** had a homogenous binding pattern to both catalytic sites of the enzymes, in terms of the conformations found in the same 2 Å RMSD cluster. The *para-*substituents from both the aromatic rings are important for the interaction of the compounds with the COX binding sites. The CF_3_ substituent from **5c** formed hydrogen bonds with Ser532 from COX-1 and Tyr371 from COX-2. The interaction with the serine amino acid residue from COX-1 is important to COX-1 inhibition [42].

The antinociceptive activity evaluated in a model of inflammatory pain showed an increase in pain threshold for all tested compounds when compared to negative control group. Compounds **5g** (*p*ara-Cl and unsubstituted phenyl), **5i** (*para*-OCH_3_ and unsubstituted phenyl) and **5j** (*para*-OCH_3_ and *para*-Br) presented a significant increase in pain threshold compared to negative control group. Analgesic activity studies revealed that compounds with unsubstituted phenyl and with *para*-bromophenyl and *para*-fluorophenyl exhibited good analgesic activities [43,44].

Most of the NSAIDs are associated with gastric side effects at the anti-inflammatory, analgesic and antipyretic dosage. The inhibition of COX-1 is associated with gastric injury. Diclofenac, a non-selective NSAID, when administered by gavage (20 mg/kg bw) had the highest ulceration score among all tested compounds, even if it is considered the most active against COX-2 among several other carboxylic acid-containing NSAIDs [7]. The ulceration risk of all synthesized compounds was significantly reduced when compared to diclofenac group, giving them a good safety profile.

## 4. Materials and Methods

### 4.1. Chemistry

#### 4.1.1. Reagents and Solvents

All the commercially available chemicals necessary for the synthesis were purchased from Sigma-Aldrich Chemie GmbH (Steinheim, Germany), TCI Europe N.V. (Zwijndrecht, Belgium) or Merck KgaA (Darmstadt, Germany) and were used as supplied, without further purification.

#### 4.1.2. Analytical Methods

Chemical reactions were monitored by thin layer chromatography (TLC) on pre-coated silica gel 60F254 sheets from Merck (Darmstadt, Germany), and dichloromethane and a mixture of dichloromethane: acetone 25:1 *v*/*v* was used as elution system. The spots were viewed in UV light at 254 nm. The compounds were recrystallized from ethanol or a mixture of ethanol: water. Preparative chromatographic purifications were performed using Merck Kieselgel 60 Å column chromatography and dichloromethane and a mixture of dichloromethane: acetone 25:1 *v*/*v* as eluent.

Melting points were determined using open capillary tube method, with an Electrothermal IA 9000 digital apparatus (Bibby Scientific Limited, Staffordshire, UK), and were uncorrected.

The MS spectra were recorded using an Agilent 1100 Ion Trap mass spectrometer (Agilent Technologies, Santa Clara, CA, USA) operating at 70 eV. ^1^H-NMR, ^13^C-NMR and ^19^F-NMR spectra were recorded on Bruker Avance DRX-600 spectrometer (Billerica, MA, USA) operating at 600 MHz, 151 MHz and 565 MHz, in trifluoroacetic acid (TFA)-*d*_6_ and acetone-*d*_6_ (external standard), dimethylsulfoxide (DMSO)-*d*_6_, deuterated chloroform (CDCl_3_), dimethylformamide (DMF)-*d*_6_, acetic acid-*d*_6_ and as solvents and tetramethylsilane (TMS) as internal standard. The spectral data are mentioned in the Materials and Methods section, using the following abbreviations for peak patterns: s-singlet, d-doublet, dd-double doublet, t-triplet, q-quartet.

The FT-IR spectra were performed on a Jasco FT/IR 470 Plus spectrometer (Easton, MD, USA) using the ATR technique. The IR spectra were recorded between 4000 and 400 cm^−1^ wavelengths at 4 cm^−1^ resolution.

#### 4.1.3. Synthesis of Imidazo[2,1-*b*][1,3,4]thiadiazole Derivatives

Synthesis of 5-(4-substituted phenyl)-1,3,4-thiadiazole-2-amine **3a**–**l**

0.1 M of aromatic carboxylic acid **1a**–**l** was refluxed with 0.2 M of thiosemicarbazide ***2*** and 5 mL of phosphorous oxytrichloride for 2 h. After 2 h the mixture was cooled and diluted with 10 mL of water and again refluxed for additional 4 h. Then the mixture was filtered hot and the filtrate was neutralized with a solution of 10% potassium hydroxide. The formed precipitate was filtered off and recrystallized from absolute ethanol.

General procedure for the synthesis of 6-(4-substituted phenyl)-2-(4-substituted phenyl)imidazo[2,1-*b*][1,3,4]thiadiazole **5a**–**l**

To a solution of 5-(4-substituted phenyl)-1,3,4-thiadiazole-2-amine **3a**–**l** (0.01 mol) in 10 mL absolute ethanol, the corresponding phenacyl bromide was added (0.02 mol). The mixture was refluxed for 18 h or more, depending of the reaction progress, monitored by TLC. After the reaction was complete, the mixture was filtered and the solid hydrobromide was collected. The precipitate was neutralized by aqueous sodium carbonate solution, filtered and purified by recrystallization or by column chromatography to remove unwanted products. The purification method is indicated below for each individual compound.

2,6-Diphenylimidazo[2,1-*b*][1,3,4]thiadiazole (**5a**) [28]: Yield 53% (0.146 g); purified by column chromatography, eluent dichloromethane: acetone 25:1 *v*/*v*; m.p. 200–202 °C; cristaline white powder; FT-IR (solid state, υ cm^−1^): 3094, 3049, 3026 (CH aromatic); ^1^H-NMR (600 MHz, DMSO-*d*_6_, CDCl_3_) δ 8.53 (s, 1H, CH-5 imidazo[2,1-*b*][1,3,4]thiadiazole), 7.93 (dd, *J* = 7.6, 2H, CH-2, CH-6), 7.88 (d, *J* = 7.2 Hz, 2H, CH-2’, CH-6’), 7.62–7.53 (m, 3H, CH-4, CH-3’, CH-5’), 7.40 (t, *J* = 7.7 Hz, 2H, CH-3, CH-5), 7.28 (t, *J* = 7.4 Hz, 1H, CH-4’); ^13^C-NMR (151 MHz, DMSO-*d*_6_, CDCl_3_) δ 151.13 (C, C-2 imidazo[2,1-*b*][1,3,4]thiadiazole), 135.85 (C, C-8 imidazo[2,1-*b*][1,3,4]thiadiazole), 134.54 (C, C-6 imidazo[2,1-*b*][1,3,4]thiadiazole), 123.85 (C, C-1), 121.82 (C, C-1’), 119.87 (C, C-4), 119.43 (C, C-3’, C-5’), 118.61 (C, C-3, C-5), 117.36 (C, C-2’, C-6’), 116.65 (C, C-4’), 114.85 (C, C-2, C-6), 100.25 (C, C-5 imidazo[2,1-*b*][1,3,4]thiadiazole); ESI^+^-MS: *m/z* 278.2 [M + H]^+^ (calcd. 278.3 for C_16_H_11_N_3_S + H^+^).

6-(4-Bromophenyl)-2-phenylimidazo[2,1-*b*][1,3,4]thiadiazole (**5b**) **[27,28]**: Yield 40% (0.140 g); m.p. 240–242 °C; yellow powder; FT-IR (solid state, υ cm^−1^): 3095, 3071, 3051 (CH aromatic); ^1^H-NMR (600 MHz, TFA-*d*_6_, acetone-*d*_6_) δ 7.89 (s, 1H, CH-5 imidazo[2,1-*b*][1,3,4]thiadiazole), 7.41 (d, *J* = 8.5 Hz, 2H, CH-2, CH-6), 7.23 (m, *J* = 7.9, 3.8 Hz, 4H, CH-2’, CH-3’, CH-5’ CH-6’), 7.08 (t, *J* = 7.5 Hz, 1H, CH-4), 6.99 (t, *J* = 7.7 Hz, 2H, CH-3, CH-5); ^13^C-NMR (151 MHz, TFA-*d*_6_, acetone-*d*_6_) δ 157.60 (C, C-2 imidazo[2,1-*b*][1,3,4]thiadiazole), 157.33 (C, C-8 imidazo[2,1-*b*][1,3,4]thiadiazole), 156.89 (C, C-6 imidazo[2,1-*b*][1,3,4]thiadiazole), 132.47 (C, C-1), 132.06 (CH, C-3’, C-5’), 131.56 (CH, C-4), 130.44 (C, C-1’), 128.99 (CH, C-3, C-5), 128.62 (CH, C-2’, C-6’), 125.75 (CH, C-2, C-6), 125.52 (C, C-4’), 115.86 (C, C-5 imidazo[2,1-*b*][1,3,4]thiadiazole); ESI^+^-MS: *m/z* 356.2 ([M + H]^+^, ^79^Br), 358.2 ([M + H]^+^, ^81^Br) (calcd. for C_16_H_10_BrN_3_S + H^+^: 356.1 ^79^Br, 358.0 ^81^Br).

2-Phenyl-6-(4-(trifluoromethyl)phenyl)imidazo[2,1-*b*][1,3,4]thiadiazole (**5c**): Yield 38% (0.130 g); purified by column chromatography, eluent dichloromethane; m.p. 218–220 °C; yellow powder; FT-IR (solid state, υ cm^−1^): 3107, 3084 (CH aromatic); ^1^H-NMR (600 MHz, TFA-*d*_6_, acetone-*d*_6_) δ 8.21 (s, 1H, CH-5 imidazo[2,1-*b*][1,3,4]thiadiazole), 7.83 (t, *J* = 8.0 Hz, 2H, CH-2, CH-6), 7.71 (m, *J* = 8.4 Hz, 4H, CH-2’, CH-3’, CH-5’, CH-6’), 7.55 (t, *J* = 8.5 Hz, 1H, CH-4), 7.48–7.42 (m, 2H, CH-3, CH-5); ^13^C-NMR (151 MHz, TFA-*d*_6_, acetone-*d*_6_) δ 172.26 (C, C-2 imidazo[2,1-*b*][1,3,4]thiadiazole), 161.09 (C, C-8 imidazo[2,1-*b*][1,3,4]thiadiazole), 147.89 (C, C-6 imidazo[2,1-*b*][1,3,4]thiadiazole), 140.75 (C, C-1′), 136.89 (q, *J*_2C-F_ = 34.7 Hz, C, C-4′), 133.22 (CH, C-3, C-5), 132.23 (CH, C-4), 132.04 (C, C-1), 130.74 (q, *J*_3C-F_ =3 Hz, CH, C-3’, C-5’), 130.19 (CH, C-2, C-6), 129.76 (CH, C-2’, C-6’), 126.9 (q, *J*_1C-F_ = 272 Hz, C, CF_3_), 115.65 (C, C-5 imidazo[2,1-*b*][1,3,4]thiadiazole); ^19^F-NMR (565 MHz, TFA-*d*_6_, acetone-*d*_6_): δ -64.05 (s, -CF_3_); ESI^+^-MS: *m/z* 346.3 [M + H]^+^ (calcd. 346.3 for C_17_H_10_F_3_N_3_S + H^+^).

6-(4-Methoxyphenyl)-2-phenylimidazo[2,1-*b*][1,3,4]thiadiazole (**5d**) [16,28]: Yield 27% (0.080 g); m.p. 232–234 °C; gray powder; FT-IR (solid state, υ cm^−1^): 3107, 3069 (CH aromatic); ^1^H-NMR (600 MHz, DMF-*d*_6_) δ 8.93 (s, 1H, CH-5 imidazo[2,1-*b*][1,3,4]thiadiazole), 8.08 (d, *J* = 6.9 Hz, 2H, CH-2, CH-6), 7.98 (d, *J* = 8.8 Hz, 2H, CH-2’, CH-6’), 7.73–7.64 (m, 3H, CH-3, CH-4, CH-5) 7.10 (d, *J* = 8.8 Hz, 2H, CH-3’, CH-5’), 3.87 (s, 3H, CH_3_); ^13^C-NMR (151 MHz, DMF-*d*_6_) δ 164.03 (C, C-2 imidazo[2,1-*b*][1,3,4]thiadiazole), 160.46 (C, C-4’), 144.50 (C, C-8 imidazo[2,1-*b*][1,3,4]thiadiazole ), 142.45 (C, C-6 imidazo[2,1-*b*][1,3,4]thiadiazole), 132.88 (C, C-1), 130.03 (CH, C-4), 129.45 (CH, C-3, C-5), 127.39 (CH, C-2’, C-6’), 127.03 (CH, C-2, C-6), 123.49 (C, C-1’), 114.75 (C, C-5 imidazo[2,1-*b*][1,3,4]thiadiazole), 110.51 (C, C-3’, C-5’), 55.40 (C, OCH_3_); ESI^+^-MS: *m/z* 308.3 [M + H]^+^ (calcd. 308.3 for C_17_H_13_N_3_OS + H^+^).

2-(4-Chlorophenyl)-6-phenylimidazo[2,1-*b*][1,3,4]thiadiazole (**5e**): Yield 32% (0.100 g); m.p. 220–222 °C; gray powder; FT-IR (solid state, υ cm^−1^): 3125, 3096 (CH aromatic); ^1^H-NMR (600 MHz, TFA-*d*_6_, acetone-*d*_6_) δ 8.42 (s, 1H, CH-5 imidazo[2,1-*b*][1,3,4]thiadiazole), 8.10 (d, 2H, CH-2, CH-6), 7.87 (d, 2H, CH-2’, CH-6’), 7.77–7.72 (m, 5H, CH-3, CH-4, CH-5, CH-3’, CH-5’); ^13^C-NMR (151 MHz, TFA-*d*_6_, acetone-*d*_6_) δ 168.84 (C, C-2 imidazo[2,1-*b*][1,3,4]thiadiazole), 145.47 (C, C-8 imidazo[2,1-*b*][1,3,4]thiadiazole), 142.94 (C, C-6 imidazo[2,1-*b*][1,3,4]thiadiazole), 141.72 (C, C-4), 133.06 (C, C-1), 131.82 (CH, C-3, C-5), 131.32 (CH, C-3’, C-5’), 130.02 (CH, C-2, C-6), 127.38 (CH, C-2’, C-6’), 127.07 (C, C-1’), 126.63 (C, C-4’), 112.58 (CH, C-5 imidazo[2,1-*b*][1,3,4]thiadiazole); ESI^+^-MS: *m/z* 312.6 ([M + H]^+^, ^35^Cl), 314.6 ([M + H]^+^, ^37^Cl) (calcd. for C_16_H_10_ClN_3_S + H^+^: 312.8 ^35^Cl, 314.8 ^37^Cl).

6-(4-Bromophenyl)-2-(4-chlorophenyl)imidazo[2,1-*b*][1,3,4]thiadiazole (**5f**) [27,28]: Yield 77% (0.230 g); purified by column chromatography, eluent dichloromethane; m.p. 256–258 °C; gray powder; FT-IR (solid state, υ cm^−1^): 3119, 3071, 3047 (CH aromatic); ^1^H-NMR (600 MHz, TFA-*d*_6_, acetone-*d*_6_) δ 8.11 (s, 1H, CH-5 imidazo[2,1-*b*][1,3,4]thiadiazole), 7.77 (d, *J* = 8.4 Hz, 2H, CH-2, CH-6), 7.57 (d, *J* = 8.3 Hz, 2H, CH-2’, CH-6’), 7.45 (d, *J* = 8.4 Hz, 2H, CH-3, CH-5), 7.41 (d, *J* = 8.3 Hz, 2H, CH-3’, CH-5’); ^13^C-NMR (151 MHz, TFA-*d*_6_, acetone-*d*_6_) δ 168.82 (C, C-2 imidazo[2,1-*b*][1,3,4]thiadiazole), 145.55 (C, C-8 imidazo[2,1-*b*][1,3,4]thiadiazole), 142.94 (C, C-6 imidazo[2,1-*b*][1,3,4]thiadiazole), 140.58 (C, C-4), 134.55 (CH, C-3’, C-5’), 131.65 (CH, C-3, C-5), 129.78 (CH, C-2’, C-6’), 128.54 (CH, C-2, C-6), 127.62 (C, C-1), 126.72 (C, C-1’), 125.64 (C, C-4’), 116.28 (CH, C-5 imidazo[2,1-*b*][1,3,4]thiadiazole); ESI^+^-MS: *m/z* 390.2 ([M + H]^+^, ^79^Br), 392.2 ([M + H]^+^, ^81^Br) (calcd. for C_16_H_9_BrClN_3_S + H^+^: 390.6 ^79^Br, 392.6 ^81^Br).

2-(4-Chlorophenyl)-6-(4-(trifluoromethyl)phenyl)imidazo[2,1-*b*][1,3,4]thiadiazole (**5g**): Yield 54% (0.200 g); purified by column chromatography, eluent dichloromethane; m.p. 228–230 °C; yellow powder; FT-IR (solid state, υ cm^−1^): 3104, 2922 (CH aromatic); ^1^H-NMR (600 MHz, TFA-*d*_6_, acetone-*d*_6_) δ 8.20 (s, 1H, CH-5 imidazo[2,1-*b*][1,3,4]thiadiazole), 7.77 (d, *J* = 7.0 Hz, 2H, CH-3’, CH-5’), 7.69–7.67 (m, 4H, CH-2, CH-3, CH-5, CH-6), 7.43 (d, *J* = 6.7 Hz, 2H, CH-2’, CH-6’); ^13^C-NMR (151 MHz, TFA-*d*_6_, acetone-*d*_6_) δ 170.88 (C, C-2 imidazo[2,1-*b*][1,3,4]thiadiazole), 147.89 (C, C-8 imidazo[2,1-*b*][1,3,4]thiadiazole), 144.66 (C, C-6 imidazo[2,1-*b*][1,3,4]thiadiazole), 141.73 (C, C-4) 136.89 (q, *J*_2C-F_ = 34.7 Hz, C, C-4′), 133.63 (CH, C-3, C-5), 132.03 (C, C-1), 131.92 (CH, C-2, C-6), 130.11 (q, *J*_3C-F_ = 3 Hz, CH, C-3′, C-5′), 129.81 (CH, C-2′, C-6′ ), 128.77 (C, C-1′), 127.07 (q, *J*_1C-F_ = 272 Hz, C, CF_3_), 115.71 (CH, C-5 imidazo[2,1-*b*][1,3,4]thiadiazole); ^19^F-NMR (565 MHz, TFA-*d*_6_, acetone-*d*_6_): δ -64.05 (s, -CF_3_); ESI^+^-MS: *m/z* 380.2 ([M + H]^+^, ^35^Cl), 382.2 ([M + H]^+^, ^37^Cl) (calcd. for C_17_H_9_ClF_3_N_3_S + H^+^: 380.7 ^35^Cl, 382.7 ^37^Cl).

2-(4-Chlorophenyl)-6-(4-methoxyphenyl)imidazo[2,1-*b*][1,3,4]thiadiazole (**5h**) [8,16,27]: Yield 44% (0.150 g); purified by column chromatography, eluent dichloromethane: acetone 25:1 *v*/*v*; m.p. 228–230 °C; gray powder; FT-IR (solid state, υ cm^−1^): 3119, 3086 (CH aromatic); ^1^H-NMR (600 MHz, TFA-*d*_6_, acetone-*d*_6_) δ 8.02 (s, 1H, CH-5 imidazo[2,1-*b*][1,3,4]thiadiazole), 7.75 (d, *J* = 8.2 Hz, 2H, CH-2’, CH-6’), 7.51 (d, *J* = 8.4 Hz, 2H, CH-2, CH-6), 7.42 (d, *J* = 8.2 Hz, 2H, CH-3’, CH-5’), 7.02 (d, *J* = 8.4 Hz, 2H, CH-3, CH-5), 3.84 (s, 3H, CH_3_); ^13^C-NMR (151 MHz, TFA-*d*_6_, acetone-*d*_6_) δ 168.55 (C, C-2 imidazo[2,1-*b*][1,3,4]thiadiazole), 162.39 (C, C-4’), 145.02 (C, C-8 imidazo[2,1-*b*][1,3,4]thiadiazole), 142.82 (C, C-6 imidazo[2,1-*b*][1,3,4]thiadiazole), 141.17 (C, C-4), 131.67 (CH, C-3, C-5), 129.82 (CH, C-2’, C-6’), 129.24 (CH, C-2, C-6), 126.90 (C, C-1), 120.45 (C, C-1), 117.03 (CH, C-3’, C-5’), 111.94 (CH, C-5 imidazo[2,1-*b*][1,3,4]thiadiazole), 56.82 (C, OCH_3_); ESI^+^-MS: *m/z* 342.6 ([M + H]^+^, ^35^Cl), 344.6 ([M + H]^+^, ^37^Cl) (calcd. for C_17_H_12_ClN_3_OS + H^+^: 342.8 ^35^Cl, 344.8 ^37^Cl).

2-(4-Methoxyphenyl)-6-phenylimidazo[2,1-*b*][1,3,4]thiadiazole (**5i**) [26]: Yield 74% (0.230 g); m.p. 198–200 °C; yellow powder; FT-IR (solid state, υ cm^−1^): 3127, 3071 (CH aromatic); ^1^H-NMR (600 MHz, Acetic acid) δ 8.28 (s, 1H, CH-5 imidazo[2,1-*b*][1,3,4]thiadiazole), 7.94 (d, *J* = 7.7 Hz, 2H, CH-2, CH-6), 7.82 (d, *J* = 6.7 Hz, 2H, CH-2’, CH-6’), 7.47 (t, *J* = 6.6 Hz, 2H, CH-3’, CH-5’), 7.39 (t, *J* = 6.4 Hz, 1H, CH-4’), 7.12 (d, *J* = 7.9 Hz, 2H, CH-3, CH-5), 3.91 (s, 3H, OCH_3_); ^13^C-NMR (151 MHz, Acetic acid) δ 166.00 (C, C-2 imidazo[2,1-*b*][1,3,4]thiadiazole), 164.57 (C, C-4), 146.96 (C, C-8 imidazo[2,1-*b*][1,3,4]thiadiazole), 145.30 (C, C-6 imidazo[2,1-*b*][1,3,4]thiadiazole), 133.28 (C, C-1’), 130.54 (CH, C-3’, C-5’), 130.25 (C, C-2’, C-6’), 129.90 (C, C-1), 126.97 (C, C-2, C-6), 123.68 (CH, C-4’), 116.39 (C, C-3, C-5), 111.73 (CH, C-5 imidazo[2,1-*b*][1,3,4]thiadiazole), 56.62 (C, OCH_3_); ESI^+^-MS: *m/z* 308.4 [M + H]^+^ (calcd. 308.3 for C_17_H_13_N_3_OS + H^+^).

6-(4-Bromophenyl)-2-(4-methoxyphenyl)imidazo[2,1-*b*][1,3,4]thiadiazole (**5j**) [26]: Yield 74% (0.280 g); purified by column chromatography, eluent dichloromethane: acetone 25:1 *v*/*v*; m.p. 224–225 °C; yellow powder; FT-IR (solid state, υ cm^−1^): 3107, 3084 (CH aromatic); ^1^H-NMR (600 MHz, DMSO-*d*_6_) δ 8.79 (s, 1H, CH-5 imidazo[2,1-*b*][1,3,4]thiadiazole), 7.90 (d, 2H, CH-2, CH-6), 7.84 (d, 2H, CH-3’, CH-5’), 7.63 (d, 2H, CH-3, CH-5), 7.16 (d, 2H, CH-2’, CH-6’), 3.86 (s, 3H, OCH_3_); ^13^C-NMR (151 MHz, DMSO-*d*_6_) δ 162.22 (C, C-2 imidazo[2,1-*b*][1,3,4]thiadiazole), 161.40 (C, C-4), 144.50 (C, C-8 imidazo[2,1-*b*][1,3,4]thiadiazole), 144.07 (C, C-6 imidazo[2,1-*b*][1,3,4]thiadiazole), 133.14 (C, C-1’), 131.64 (CH, C-3’, C-5’), 128.48 (CH, C-2’, C-6’), 126.62 (CH, C-2, C-6), 121.94 (C, C-1), 120.18 (C, C-4’), 115.05 (CH, C-3, C-5), 111.06 (CH, C-5 imidazo[2,1-*b*][1,3,4]thiadiazole), 55.64 (C, OCH_3_); ESI^+^-MS: *m/z* 386.1 ([M + H]^+^, ^79^Br), 388.1 ([M + H]^+^, ^81^Br) (calcd. for C_16_H_9_BrClN_3_S + H^+^: 386.2 ^79^Br, 388.2 ^81^Br).

2-(4-Methoxyphenyl)-6-(4-(trifluoromethyl)phenyl)imidazo[2,1-*b*][1,3,4]thiadiazole (**5k**): Yield 59% (0.220 g); m.p. 228–230 °C; yellow powder; FT-IR (solid state, υ cm^−1^): 3087, 3024 (CH aromatic); ^1^H-NMR (600 MHz, TFA-*d*_6_, acetone-*d*_6_) δ 8.16 (s, 1H, CH-5 imidazo[2,1-*b*][1,3,4]thiadiazole), 7.80 (d, *J* = 8.8 Hz, 2H, CH-2, CH-6), 7.66-7.69 (m, 4H, CH-2’, CH-3’, CH-5’, CH-6’), 7.01 (d, *J* = 8.8 Hz, 2H, CH-3, CH-5), 3.84 (s, 3H, OCH_3_); ^13^C-NMR (151 MHz, TFA-*d*_6_, acetone-*d*_6_) δ 171.58 (C, C-2 imidazo[2,1-*b*][1,3,4]thiadiazole), 167.09 (C, C-8 imidazo[2,1-*b*][1,3,4]thiadiazole), 147.68 (C, C-6 imidazo[2,1-*b*][1,3,4]thiadiazole), 141.32 (C, C-4), 136.86 (q, *J*_2C-F_ = 34.7 Hz, C, C-4’), 132.86 (C, C-2, C-6), 132.14 (C, C-1), 130.10 (q, *J*_3C-F_ = 3 Hz, C, C-3’, C-5’), 129.79 (C, C-2’, C-6’), 127.09 (q, *J*_1C-F_ = 270.3 Hz, C, CF_3_), 123.39 (C, C-1′), 118.84 (C, C-3, C-5), 115.59 (CH, C-5 imidazo[2,1-*b*][1,3,4]thiadiazole), 58.65 (C, OCH_3_); ^19^F-NMR (565 MHz, TFA-*d*_6_, acetone-*d*_6_): δ -64.03 (s, -CF_3_); ESI^+^-MS: *m/z* 376.4 [M + H]^+^ (calcd. 376.3 for C_18_H_12_F_3_N_3_OS + H^+^).

2,6-Bis(4-methoxyphenyl)imidazo[2,1-*b*][1,3,4]thiadiazole (**5l**) [26]: Yield 60% (0.200 g); purified by column chromatography, eluent dichloromethane; m.p. 242–244 °C; yellow powder; FT-IR (solid state, υ cm^−1^): 3124, 3047 (CH aromatic); ^1^H-NMR (600 MHz, CDCl_3_) δ 7.94 (s, 1H, CH-5 imidazo[2,1-*b*][1,3,4]thiadiazole), 7.82 (d, *J* = 8.8 Hz, 2H, CH-2, CH-6), 7.77 (d, *J* = 8.7 Hz, 2H, CH-2’, CH-6’), 7.03 (d, *J* = 8.8 Hz, 2H, CH-3, CH-5), 6.98 (d, *J* = 8.7 Hz, 2H, CH-3’, CH-5’), 3.89 (s, 3H, CH_3_), 3.83 (s, 3H, OCH_3_); ^13^C-NMR (151 MHz, CDCl_3_) δ 164.73 (C, C-2 imidazo[2,1-*b*][1,3,4]thiadiazole), 163.21 (C, C-4), 160.50 (C, C-4’), 150.09 (C, C-8 imidazo[2,1-*b*][1,3,4]thiadiazole) 143.69 (C, C-6 imidazo[2,1-*b*][1,3,4]thiadiazole), 128.78 (CH, C-2’, C-6’), 126.97 (CH, C-2, C-6), 124.74 (C, C-1), 120.95 (C, C-1’), 114.98 (CH, C-3’, C-5’), 114.63 (CH, C-3, C-5), 108.42 (CH, C-5 imidazo[2,1-*b*][1,3,4]thiadiazole), 55.59 (C, OCH_3_), 55.33 (C, OCH_3_). ESI^+^-MS: *m/z* 338.4 [M + H]^+^ (calcd. 338.3 for C_17_H_13_N_3_OS + H^+^).

### 4.2. Pharmacology

#### 4.2.1. Animals

For the evaluation of the biological potential of the synthesized compounds, 14 groups of female Charles River Wistar (Crl:WI) rats (*n* = 6) weighing 180–220 g were purchased from the Practical Skills and Experimental Medicine Centre of the ”Iuliu Hațieganu” University of Medicine and Pharmacy (Cluj-Napoca, Romania). The animals were housed in polycarbonate type IV-S open-top cages (Tecniplast, Italy) and kept under controlled room temperature (22 ± 2 °C; relative humidity 45% ± 10%,) with a 12/12 h light/dark cycle. The animals were fed with standard pelleted diet (Cantacuzino Institute, Bucharest, Romania) and received water ad libitum. Food was withdrawn 24 h before experiments. All animal procedures were performed in accordance with the EEC Directive 63/2010, which regulates the care and use of laboratory animals for scientific purposes and were approved by The Sanitary-Veterinary and Food Safety Directorate from Cluj (66/06.06.2017).

The animals were divided into groups (*n* = 6) and 12 synthesized compounds were solubilized in 0.5% Tween 80 solution and administered in dose of 50 mg/kg bw by gavage to the corresponding groups. The negative control group received the vehicle (0.5% Tween 80 solution, by gavage) and the control positive group was treated with diclofenac sodium (Gerot Lannach GmbH, Lannach, Austria) (20 mg/kg bw, by gavage) as reference drug.

#### 4.2.2. Anti-Inflammatory Activity

Anti-inflammatory activity was determined by λ-carrageenan-induced rat paw edema test [29,45]. Acute inflammation was induced 1 h after the intragastric administration of substances, by intraplantar injection of 100 μL of 1% λ-carrageenan (Sigma Aldrich, St. Louis, MO, USA) saline solution into left hind paw of the rats. The paw volume (mL) was measured plethysmometrically (Ugo Basile 7140, Varese, Italy) at 0, 1, 2, 3 and 4 h after λ-carrageenan injection. Edema volume and the percentage of edema inhibition were expressed as follows:

Edema volume (mL) = V_t_ − V_0_ (1)

Inhibition of edema (%) = [1 − (Et/Ec) × 100]
(2)

where V_0_ is the mean paw volume before λ-carrageenan intraplantar injection, V_t_ is the mean paw volume at ”t” hours, Et is mean edema volume in treated animals and Ec is mean edema volume in the control group.

#### 4.2.3. Antinociceptive Activity

The nociceptive withdrawal threshold was assessed by Randall-Selitto test [30,46]. For this experiment, to reduce the number of animals, the same groups of rats previously tested for anti-inflammatory potential were used. In this model of inflammatory pain, the pain threshold of the inflamed hind paw of the rats was determined at 0, 1, 2, 3 and 4 h after the λ-carrageenan intraplantar injection, using an analgesimeter (Ugo Basile 37215, Varese, Italy). The analgesimeter applied a linearly increased force (grams) until the animal produced a response characterized by removal of the paw or noises, interpreted as mechanical hypernociception. The instrument recorded the maximal amount of pressure (grams) withstood by rats at each time interval.

#### 4.2.4. Ulcerogenic Activity

Ulcerogenic activity of the synthesized compounds after a single oral administration of 50 mg/kg bw was evaluated and scored by the method of Cioli et al. [47] adapted by Assarzadeh et al. [44]. To test the ulcerogenic activity, all 14 groups (n = 6) were fasted for 24 h prior to drug administration and housed in mesh floor grid cages to avoid the coprophagy. They had access to water ad libitum. At 6 h after the substance administration, all animals were sacrificed under deep anesthesia, then their stomachs were removed, opened along the great curvature and rinsed with saline solution 0.9%. The gastric mucosa was inspected under magnifying lens (2×) to assess the incidence of redness and spot ulcers. The mucosal damage was evaluated according to the following score: 0.5: redness; 1.0: spot ulcers; 1.5: hemorrhagic streaks; 2.0: ulcers > 3 but ≤ 5; and 3.0: ulcers > 5. The gastric mucosal ulceration score was calculated by the difference between the mean score of each treated group and the mean score of control group. Diclofenac (20 mg/kg bw) was used as reference drug.

#### 4.2.5. Statistical Data Analysis

Statistics were performed with one-way analysis of variance (ANOVA), followed by Dunnett’s multi-comparison test. All data are presented as mean ± standard error or mean (SEM), *p* < 0.05 was considered to be significant.

### 4.3. Molecular Docking

In order to understand the mechanism of action of the synthesized compounds and to know the nature of interactions between the compounds and the active sites of COX, we performed a molecular docking study. Because no three-dimensional crystallographic structures of COX-1 and COX-2 isolated from *Rattus norvegicus* are available in the Protein Data Bank (www.rcsb.org), these structures were built by homology modeling using Swiss-Model [48]. The primary amino acid sequences of both proteins were retrieved from Uniprot (www.uniprot.org). The construction of the chimeric COX-1 was made using the Q63921 sequence and the PDB three-dimensional structure 1CQE as template, with 96% coverage and 85.94% identity, referred to as the primary amino acid sequence. The chimeric COX-2 structure was built using the P35355 sequence and the 4RRX PDB structure, with which it has 97% coverage and 96.08% identity. The sequence alignment between both Q63921 and P35355 primary amino acid sequences and the corresponding templates 1CQE, respectively 4RRX used for homology modeling are presented in Supplementary Materials (Figures S1 and S2).

The ligands and the constructed chimeric structures COX-1 and COX-2 were further handled to prepare them for the molecular docking study as targets using AutoDock Tools 1.5.6 [49]. The preparation followed the general protocol, represented by the protonation of amines, the deprotonation of carboxylic acids and the addition of the Gasteiger partial charges [50].

The search space was set as cubic for both enzymes, with edges set equal to 50 Å, resulting in 125.000 Å^3^, with a spacing set to 0.375 Å. The Cartesian coordinates of the center of the search space were set to x = 27.805, y = 35.244, z = 207.085 for COX-1, respectively x = 26.131, y = −40.62, z = −16.423 for COX-2. Search space was defined relative to the template structures, to include the crystallized inhibitors found in the templates used in modeling. In addition, the sequences of the built COX-1 and COX-2 chimeric structures were analyzed using blast [51], to obtain information about the substrate binding sites and to ensure the inclusion of the catalytic residues into the docking search space.

For each compound, 50 conformations were generated using AutoDock 4.2 [49]. The results of the docking study were ranked in descending order, according to the calculated binding energies (∆G) and grouped in clusters with 2 Å RMSD tolerance of the atoms’ coordinates.

The binding interactions between ligands and targets were depicted using UCSF Chimera 1.10.2 [52]. Sequence alignment between FASTA sequences was performed using Clustal Omega [53]. Diclofenac was docked as reference.

## 5. Conclusions

In the present study we report the synthesis, characterization and anti-inflammatory/analgesic evaluation of 2,6-diaryl-imidazo[2,1-*b*][1,3,4]thiadiazole derivatives **5a**–**l**. The spectral analysis (IR, ^1^H-NMR, ^13^C-NMR, ^19^F-NMR and MS) confirmed the structures of the desired compounds. The anti-inflammatory activity of these compounds was evaluated in vivo, in a model of acute inflammation induced by λ-carrageenan in rats; molecular docking studies were made to elucidate the potential mechanism of anti-inflammatory activity. The results of the present study showed that in vivo anti-inflammatory studies are in accordance with in silico studies and the compound **5c** which showed the best anti-inflammatory activity, presented the highest COX-2 inhibition. Compounds **5g**, **5i** and **5j** presented significant analgesic properties. All compounds showed significantly lower ulcerogenic risk than the reference drug, diclofenac. These compounds are good candidates for further investigations regarding their potential use in therapy for the treatment of pain and inflammatory diseases.

## Figures and Tables

**Table 1:** In vivo anti-inflammatory activity of synthesized compounds on carrageenan-induced paw edema in rats.

Compound	Dose (mg/kg) p.o.	Edema 1 h (mL)	Edema 2 h (mL)	Edema 3 h (mL)	Edema 4 h (mL)
		(%inhib.)	(%inhib.)	(%inhib.)	(%inhib.)
Negative control (vehicle)	-	1.38 ± 0.15	2.22 ± 0.24	3.05 ± 0.18	3.20 ± 0.25
**5a**	50	1.33 ± 0.05	1.98 ± 0.20	2.52 ± 0.19	2.42 ± 0.24 *
		(3.50)	(10.79)	(17.63)	(24.40)
**5b**	50	1.09 ± 0.26	1.95 ± 0.07	2.45 ± 0.15 *	2.38 ± 0.20 *
		(20.87)	(12.37)	(19.92)	(25.60)
**5c**	50	1.00 ± 0.14	1.52 ± 0.14 *	2.29 ± 0.04 *	2.32 ± 0.16 *
		(27.50)	(31.63)	(24.95)	(27.53)
**5d**	50	1.21 ± 0.14	1.95 ± 0.07	2.82 ± 0.04	2.84 ± 0.10
		(12.55)	(12.29)	(7.81)	(11.05)
**5e**	50	0.86 ± 0.14	1.85 ± 0.12	2.69 ± 0.13	2.64 ± 0.18
		(37.88)	(16.72)	(11.90)	(17.41)
**5f**	50	1.18 ± 0.18	1.93 ± 0.20	2.67 ± 0.26	2.66 ± 0.20
		(14.60)	(13.42)	(12.66)	(16.84)
**5g**	50	1.23 ± 0.12	1.76 ± 0.15	2.66 ± 0.14	2.58 ± 0.15
		(10.86)	(20.84)	(12.99)	(19.45)
**5h**	50	0.92 ± 0.17	2.09 ± 0.18	2.63 ± 0.14	2.03 ± 0.17 *
		(33.78)	(6.00)	(13.86)	(28.05)
**5i**	50	1.01 ± 0.16	1.97 ± 0.16	2.62 ± 0.26	2.35 ± 0.25 *
		(26.90)	(11.39)	(14.19)	(26.59)
**5j**	50	0.95 ± 0.15	1.81 ± 0.14	2.67 ± 0.19	2.32 ± 0.20 *
		(31.10)	(18.59)	(12.66)	(27.53)
**5k**	50	1.27 ± 0.11	2.07 ± 0.09	2.59 ± 0.10	2.60 ± 0.19
		(8.08)	(7.05)	(15.34)	(18.61)
**5l**	50	1.10 ± 0.07	1.99 ± 0.08	2.57 ± 0.06	2.74 ± 0.15
		(20.63)	(10.64)	(15.99)	(14.23)
Diclofenac (reference drug)	20	1.21 ± 0.17	1.46 ± 0.16 *	1.95 ± 0.12 *	2.34 ± 0.10 *
		(12.30)	(34.33)	(36.03)	(26.96)

* Statistically significant, *p* < 0.05; Values are expressed as mean ± SEM; %inhib.—Edema inhibition; p.o.—per os (oral administration).

**Table 2:** Nociceptive threshold after the oral administration of tested compounds (Randall-Selitto test).

Group	Dose (mg/kg) p.o.	Paw Pressure (g)			
		1 h	2 h	3 h	4 h
Negative control (vehicle)	-	94.17± 7.57	78.33 ± 5.73	64.17 ± 2.71	44.17 ± 1.54
**5a**	50	98.33 ± 5.27	79.17 ± 3.75	70.83 ± 4.54	52.50 ± 1.71
**5b**	50	107.5 ± 6.16	87.50 ± 5.59	70.00 ± 5.63	57.50 ± 4.03 *
**5c**	50	122.50 ± 3.10 *	84.17 ± 2.39	74.17 ± 2.39	58.33 ± 2.79 *
**5d**	50	97.50 ± 5.28	79.17 ± 1.54	64.17 ± 3.01	53.33 ± 2.11
**5e**	50	113.33 ± 7.15	86.67 ± 6.54	70.83 ± 3.96	62.50 ± 3.10 *
**5f**	50	114.17 ± 4.55	87.50 ± 5.28	72.50 ± 4.03	63.33 ± 4.22 *
**5g**	50	125.83 ± 6.76 *	**100.00 ± 8.56** *	90.83 ± 6.38 *	80.00 ± 5.16 *
**5h**	50	110.83 ± 7.00	92.50 ± 5.88	76.67 ± 4.22	65.83 ± 3.01 *
**5i**	50	130.83 ± 8.41 *	98.33 ± 4.94 *	87.50 ± 4.43 *	78.33 ± 3.33 *
**5j**	50	130.00 ± 8.46 *	103.33 ± 4.94 *	90.83 ± 4.90 *	77.50 ± 2.81 *
**5k**	50	111.67 ± 4.22	85.83 ± 3.01	74.17 ± 1.54	68.33 ± 1.67 *
**5l**	50	113.33 ± 4.01	85.83 ± 3.01	75.00 ± 1.83	68.33 ± 1.05 *
Diclofenac (reference drug)	20	124.17 ± 5.07 *	107.50 ± 4.61 *	94.17 ± 5.83 *	78.33 ± 4.77 *

* Statistically significant, *p* < 0.05. Values are expressed as mean ± SEM; p.o.—per os (oral administration).

**Table 3:** Ulcerogenic activity screening of the synthesized compounds.

Group	Dose (mg/kg) p.o.	Ulceration Score
Negative control (vehicle)	-	0.00 ± 0.00
**5a**	50	0.33 ± 0.21
**5b**	50	0.08 ± 0.08
**5c**	50	0.08 ± 0.08
**5d**	50	0.17 ± 0.11
**5e**	50	0.17 ± 0.11
**5f**	50	0.25 ± 0.17
**5g**	50	0.17 ± 0.17
**5h**	50	0.42 ± 0.20
**5i**	50	0.17 ± 0.17
**5j**	50	0.08 ± 0.08
**5k**	50	0.00 ± 0.00
**5l**	50	0.08 ± 0.08
Diclofenac (reference drug)	20	2.58 ± 0.27 *

* Statistically significant, *p* < 0.05. Values are expressed as mean ± SEM; p.o.—per os (oral administration).

**Table 4:** The selectivity of the tested compounds (**5a**–**l**) to the COX 1 and COX 2 enzymes expressed as variation of the Gibbs free energy (ΔG) and the consequent predicted inhibition constant (*K*i).

Compound	COX-1		COX-2		*K*i_COX-2_/*K*i_COX-__1_
	∆G (kcal/mol)	*K*i (µM)	∆G (kcal/mol)	*K*i (µM)	
**5a**	−7.75	2.09	**−8.31**	**0.81**	0.39
**5b**	−7.51	3.13	**−8.36**	**0.74**	0.24
**5c**	−7.25	4.85	**−8.53**	**0.56**	**0.12**
**5d**	−6.78	10.72	−7.29	4.53	0.42
**5e**	−7.18	5.46	**−8.16**	**1.04**	**0.19**
**5f**	−6.51	16.91	−6.23	27.12	1.60
**5g**	−6.37	21.42	−6.28	24.93	1.16
**5h**	−7.14	5.84	**−8.16**	**1.04**	**0.18**
**5i**	−7.81	1.88	**−8.32**	**0.80**	0.43
**5j**	−7.32	4.31	**−8.21**	**0.96**	**0.22**
**5k**	−6.57	15.28	−6.69	12.48	0.82
**5l**	−6.53	16.35	−6.81	10.19	0.62
Diclofenac	−7.85	1.76	−8.01	1.34	0.76

ΔG—Variation of the Gibbs free energy; *K*i—Constant of inhibition.

**Table 5:** Clustering analysis of the resulted poses from the molecular docking study to the COX-1 and COX-2 enzymes.

Compound	COX-1			COX-2		
	Cluster of the Top Pose		Number of Clusters	Cluster of the Top Pose		Number of Clusters
	Conformations	Average ∆G (kcal/mol)		Conformations	Average ∆G (kcal/mol)	
**5a**	50	−7.52	1	50	−8.29	1
**5b**	47	−7.48	2	50	−8.34	1
**5c**	50	−7.01	1	50	−8.26	1
**5d**	34	−6.60	2	17	−7.18	2
**5e**	38	−7.05	2	17	−8.11	2
**5f**	42	−6.42	2	43	−6.17	3
**5g**	49	−6.15	2	38	−6.19	3
**5h**	28	−6.97	2	49	−7.93	2
**5i**	45	−7.70	2	42	−8.29	1
**5j**	25	−6.16	3	46	−8.16	2
**5k**	28	−6.29	3	32	−6.52	3
**5l**	50	−6.32	1	46	−6.55	2
Diclofenac	31	−7.56	3	33	−7.81	2

ΔG—Variation of the Gibbs free energy.

**Table 6:** The hydrogen bonds predicted to take place between compounds **5a**–**l** and COX-1 and COX-2 respectively.

Compound	COX-1		COX-2	
	Ligand	Protein	Ligand	Protein
**5a**	N/A	N/A	N (imidazole)	Ser516
**5b**	N (thiadiazole)	Tyr357	O-CH_3_	Trp373
			N (thiadiazole)	Arg106
**5c**	N (thiadiazole)	Tyr357	N (thiadiazole)	Arg106
	CF_3_	Ser532	CF_3_	Tyr371
**5d**	N/A	N/A	N/A	N/A
**5e**	N/A	N/A	N (imidazole)	Arg106
**5f**	N/A	N/A	N/A	N/A
**5g**	CF_3_	Ser532	N/A	N/A
**5h**	N/A	N/A	N (imidazole)	Arg106
**5i**	N (thiadiazole)	Tyr357	N (imidazole)	Arg106
**5j**	N/A	N/A	N (imidazole)	Arg106
**5k**	CF_3_	Tyr387	N/A	N/A
**5l**	N/A	N/A	N/A	N/A
Diclofenac	-COO^−^	Ser532	-COO^−^	Ser516
		Tyr387		Tyr371

N/A—Not available.

## Supplementary Materials

The following are available online, Figure S1: Alignment of amino acids in COX-1, Figure S2: Alignment of amino acids in COX-2.
